# Validation of Quality of Life Instruments for Cancer Patients – Colorectal Cancer (QLICP-CR) in patients with colorectal cancer in Northeast China

**DOI:** 10.1186/s12885-018-5135-6

**Published:** 2018-12-07

**Authors:** Min Liu, Wei Sun, Yuan-Yi Cai, Hua-Zhang Wu

**Affiliations:** 1 Department of Teaching and Student Affairs, Cancer Hospital of China Medical University, Shenyang, 110042 Liaoning China; 2 Department of Teaching and Student Affairs, Liaoning Cancer Hospital & Institute, Shenyang, 110042 Liaoning China; 3 Department of Social Medicine, School of Public Health, China Medical University, Shenyang, 110122 Liaoning China; 4 Department of Health Service Administration, School of Humanities and Social Science, China Medical University, No. 77 Puhe Road, Shenyang North New Area, Shenyang, 110122 Liaoning China

**Keywords:** QLICP-CR, FACT-C, Quality of life, Colorectal cancer, China

## Abstract

**Background:**

Measuring quality of life is important for cancer patients, but there are regional differences in age-standardized colorectal cancer incidence and mortality rates which may affect measurement. This study aimed to evaluate the reliability, validity and responsiveness of Quality of Life Instruments for Cancer Patients – Colorectal Cancer (QLICP-CR) in colorectal cancer patients in Northeast China, and assess its usefulness for evaluation of quality of life in these patients.

**Methods:**

From November 2016 to January 2017, 152 patients with colorectal cancer from Liaoning Cancer Hospital & Institute were surveyed three times using QLICP-CR and the Functional Assessment of Cancer Therapy – Colorectal (FACT-C) to measure their quality of life (on admission, 2–3 days later and at discharge). Reliability was evaluated by internal consistency and test–retest reliability. Validity was examined by item–domain correlation, criterion-related validity and factor construct validity analysis. Responsiveness was assessed using paired Student’s t tests and calculating standardized response mean.

**Results:**

Cronbach’s α coefficient for QLICP-CR ranged from 0.62 to 0.93. Pearson correlation and intra-class correlation coefficients for QLICP-GM, the five domains and the total scale of QLICP-CR ranged from 0.74 to 0.91 and 0.74 to 0.90. The item–domain correlation analysis showed good convergent validity and discriminant validity. Correlation analysis of domain scores between FACT-C and QLICP-CR showed good criterion-related validity. Exploratory factor analysis revealed that nine and three principal components were extracted from items in the two modules of QLICP-CR, and the contribution rate of cumulative variance was 70.21 and 72.26%. There were significant differences in quality of life between the first and the third measurements, with standardized response mean values ranging from 0.30 to 0.81.

**Conclusions:**

The QLICP-CR was a reliable, valid and sensitive instrument to measure quality of life in colorectal cancer patients in Northeast China.

## Background

Colorectal cancer (CRC) is the third most commonly diagnosed cancer worldwide [1]. According to the latest data, there were almost 1.4 million new cases and 693,900 deaths worldwide in 2012 [1], and 376,300 new cases and 191,000 deaths in China in 2015 [2]. In China, CRC is the fifth most common cancer in men and the fourth in women, and the fifth most common cause of death from cancer in both men and women [3]. It is therefore becoming a major public health problem in China.

CRC is one of the most curable cancers [4–6], and the number of people surviving it is growing [7]. It and its treatment strongly affect quality of life (QOL) [4]. The physiological and pathological changes caused by CRC and its treatment will inevitably lead to changes in the physical function, physiological function and mental state of patients. QOL is also known to be an independent predictor of survival in CRC patients [8, 9]. A number of studies have therefore focused on QOL in patients with CRC in recent years [10–14].

Several QOL instruments for patients with CRC have been developed, such as the European Organization for Research and Treatment of Cancer (EORTC) quality of life questionnaire QLQ-C30 (Core Module), QLQ-CR38 and QLQ-CR29 (Colorectal Module) [15–17], and the Functional Assessment of Cancer Therapy – Colorectal (FACT-C) [18, 19] from the Center on Outcomes, Research, and Education (CORE). Previous studies have confirmed that the Chinese versions of QLQ-C30, QLQ-CR29 and FACT-C are valuable tools to assess QOL in CRC patients [20–22], but these instruments were originally developed for English-speaking patients. The Quality of Life Instruments for Cancer Patients – Colorectal Cancer (QLICP-CR) has therefore been developed by Chinese researchers to take account of the cultural background in China [11].

QLICP-CR was shown to be a valid way to measure QOL of CRC patients in southern areas of mainland China ten years ago [11, 23]. However, the age-standardized CRC incidence and mortality rates have increased in the last ten years [24], and are different in different areas of China [24]. China is also a big country, and regional cultures vary widely. There are five main cultural regions, northeast, northwest, Beijing-Tianjin, southwest, and Tibet [25]. This regional culture difference appears in many aspects of life. For example, the diet in the northeast is more rough and salty, while the diet in the south is small, fine and sweet. People in the northeast tend to drink more on average than those in the south [26–28]. Refined grain and alcohol consumption patterns have been linked to an elevated risk of CRC [29, 30]. Cognitions and perceptions about health and illness also vary between cultures, which in turn affects the assessment of QOL [31]. QLICP-CR measures perceptions of QOL and is dependent on culture [32], so needs to be verified in different regions before application. However, to our knowledge, there has been no assessment of QLICP-CR among CRC patients in Northeast China. This study therefore carries out this assessment.

This study was designed to evaluate the reliability, validity and responsiveness of QLICP-CR, by using it three times per patient among CRC patients who were hospitalized and received treatment in Liaoning Cancer Hospital & Institute in Northeast China. We hoped that this would provide evidence of QLICP-CR’s suitability to assess QOL of CRC patients.

## Methods

### Study population

Liaoning Cancer Hospital & Institute is a provincial-level tertiary hospital (2330 beds) for cancer prevention, treatment, scientific research and teaching. It is the main hospital treating CRC in the north of China, because it hosts the Provincial Key Laboratory and Provincial Translational Medicine Center for CRC. Its diagnosis and treatment of CRC is advanced and high specification. In total, 98.55% of inpatients are from northeast China, suggesting that a study population sampled from this hospital could represent the CRC population in the region.

The inclusion criteria were (1) CRC patients who were hospitalized and received treatment; (2) patients with primary (not secondary) CRC, which had been pathologically diagnosed. The exclusion criteria were (1) patients who were under 18 years old; (2) patients with a mental illness or cognitive impairment; and (3) patients who also had another malignant tumor. Screening for mental illness or cognitive impairment used past history of the disease and the judgment of the doctor.

### Data collection procedure

This study was conducted from November 2016 to January 2017. After obtaining CRC patients’ written consent to the survey, QLICP-CR (V1.0) and the Chinese version of FACT-C (V4.0) questionnaires were distributed to all the patients, who were surveyed three times. The surveys were performed on admission, 2–3 days later, and at discharge.

### QLICP-CR instrument to indicate QOL of CRC patients

QLICP-CR (46 items) contains two modules, the Quality of Life Instruments for Cancer Patients – General Module (QLICP-GM) (32 items) and a specific domain (14 items, SCR1–SCR14). QLICP-GM is divided into four domains, physical (seven items, GPH1–GPH7), psychological (12 items, GPS1–GPS12), social (six items, GSO1–GSO6), and common symptoms and side-effects (seven items, GSS1–GSS7). Each item is rated on a five-point Likert-type scale (from “not at all” at 1, through “a little bit”, “somewhat”, and “quite a bit”, to “very much” at 5). Items that are positively stated are scored from 1 to 5 points, and negatively stated items from 5 to 1. The scoring guidelines for QLICP-CR state that the raw score for each domain is derived by summing the individual item scores, and the score for the total scale is the sum of the scores for the five domains, with higher scores indicating higher QOL. For comparison, raw scores for all domains were converted into standard scores by using the extreme difference method [11].

### FACT-C instrument to indicate QOL of CRC patients

The Chinese version of FACT-C was used alongside QLICP-CR to assess the criterion-related validity. FACT-C (36 items) contains the Functional Assessment of Cancer Therapy – Generic Scale (FACT-G, 27 items) and a colorectal cancer subscale (CCS, nine items). FACT-G is divided into four domains, covering physical well-being (PWB, seven items), social/family well-being (SWB, seven items), emotional well-being (EWB, six items) and functional well-being (FWB, seven items). Each item is rated on a five-point Likert-type scale (i.e. “Not at all”, “A little bit”, “Somewhat”, “Quite a bit”, and “Very much”) with scores from 0 to 4. Again, items that are positively stated are scored from 0 to 4 points, and negatively stated items from 4 to 0. The scoring guidelines for FACT-C were used to calculate the score of the items, domains, FACT-G and FACT-C total scale [18, 33].

### Measurement of demographic characteristics

Demographic characteristics included age, sex, marital status and education. Only one patient (0.66%) was unmarried, three (1.97%) were divorced, five (3.29%) were widowed, and one was separated. They were therefore combined into a single “other” group.

### Statistical analysis

Reliability was evaluated by internal consistency and test–retest reliability. Internal consistency was evaluated using Cronbach’s α coefficient. An α value of at least 0.7 was considered acceptable [34–36]. Test–retest reliability was assessed using paired Student’s tests, Pearson correlation analysis and intra-class correlation (ICC) analysis to compare the difference between the first and second measurements. ICC with 95% confidence interval (CI) was calculated on the basis of absolute agreement with a single measure under the two-way mixed model. A Pearson correlation coefficient greater than 0.8 was considered to provide good reliability [37]. An ICC value of at least 0.7 was considered acceptable [38].

Validity was evaluated using Pearson correlation analysis and exploratory factor analysis (EFA). Pearson correlation analysis was used to calculate the item–domain correlation of QLICP-CR and correlation of domain scores between QLICP-CR and FACT-C. Convergent validity was defined as an item–domain correlation of 0.40 or higher. Discriminant validity was defined as an item having a higher correlation with its own domain than with other domains [21, 34]. Correlation coefficients of domain scores between QLICP-CR and FACT-C were calculated to evaluate the criterion-related validity. EFA with the principal component method and varimax rotation was used to assess QLICP-CR structure [11]. Bartlett’s test of sphericity and the Kaiser-Mayer-Olkin (KMO) test were used. An initial eigenvalue above 1 was set as the criterion for factor extractions. Factor loadings above 0.50 were considered indicative of item loading [39].

Responsiveness was evaluated using paired Student’s t tests and calculating the standardized response mean (SRM) to compare the difference between the first and third measurements. SRMs of 0.2, 0.5 and 0.8 were taken as poor, moderate and good responsiveness [40].

Categorical variables were expressed as frequency and percentage. Continuous variables were expressed as means ± standard deviation (SD). SPSS16.0 was used for all statistical analyses, and a *P*-value < 0.05 was regarded as statistically significant.

## Results

This study involved 186 patients with CRC, and we received effective responses from 152 patients (effective response rate 81.72%).

### Population characteristics

The study population included 152 patients with mean age of 57.5 ± 12.24 years. In total, 79 patients (52.0%) were male, and 73 (48.0%) were female, with 142 patients (93.4%) married or cohabitating. A total of 17 (11.2%) patients were illiterate or had completed primary school, 76 (50.0%) had completed junior middle school, 30 (19.7%) had completed senior middle school, and 29 (19.1%) had completed junior college/university or above.

### Reliability

Internal consistency and test–retest reliability of QLICP-CR are shown in Table 1. Cronbach’s α coefficients for QLICP-GM and each domain of QLICP-CR ranged from 0.62 to 0.93. In total, 150 patients completed the second survey and these data were used for the test–retest reliability analysis. Test–retest reliability coefficients (*r*) for the five domains were between 0.74 and 0.91, with QLICP-GM and the total scale being 0.90 and 0.87. The ICC for all domains ranged from 0.74 to 0.90, with QLICP-GM and the total scale being 0.90 and 0.86. There were no significant differences between the first and second measurements for the scores of the five domains, the total score for QLICP-CR and the score for QLICP-GM (*P* > 0.05).

**Table 1:** Internal consistency and test–retest reliability of QLICP-CR

Domain	Internal consistency (α)(*n* = 152)	Test–retest reliability (*n* = 150)	
		Pearson correlation coefficients (*r*)	ICC (95% CI)
Physical domain (PHD)	0.78	0.88	0.88 (0.84–0.91)
Psychological domain (PSD)	0.93	0.91	0.90 (0.86–0.92)
Social domain (SOD)	0.62	0.85	0.85 (0.80–0.89)
Common symptoms and side-effects domain (SSD)	0.71	0.74	0.74 (0.66–0.81)
Specific domain (SPD)	0.87	0.82	0.80 (0.73–0.85)
Quality of Life Instruments for Cancer Patients – General Module (QLICP-GM)	0.91	0.90	0.90 (0.86–0.92)
Total scale (TOT)	–	0.87	0.86 (0.81–0.89)

Abbreviations: *ICC* intra-class correlation, *CI* confidence interval

### Validity

The item–domain correlations for QLICP-CR are shown in Table 2. Pearson correlation analysis showed that correlation coefficients between items and their own domains ranged from 0.19 to 0.90. The correlation coefficients of items with their own domains were all greater than the coefficients with other domains.

**Table 2:** The item–domain correlations for QLICP-CR (*n* = 152)

Domain	Item (brief description)	Item–domain correlation				
		PHD	PSD	SOD	SSD	SPD
Physical domain (PHD)	GPH l (Appetite)	0.76	0.38	0.22	0.35	0.11
	GPH 2 (Sleep)	0.65	0.21	0.10	0.23	0.26
	GPH 3 (Effects to Sexual life)	0.45	0.33	0.25	0.23	0.03
	GPH 4 (Effects to entertainment)	0.62	0.22	0.16	0.11	0.09
	GPH 5 (Housework)	0.78	0.25	0.21	0.20	−0.15
	GPH 6 (Ability of daily living)	0.77	0.29	0.29	0.17	−0.19
	GPH 7 (Mobility)	0.58	0.27	0.20	0.23	−0.04
Psychological domain (PSD)	GPS l (Anxiety/depression)	0.33	0.84	0.37	0.40	0.11
	GPS 2 (Dysthymia)	0.41	0.90	0.41	0.37	0.10
	GPS 3 (Dysphoria)	0.30	0.81	0.27	0.27	0.02
	GPS 4 (Disturbed by bad mood)	0.42	0.88	0.42	0.42	0.09
	GPS 5 (Worry of becoming worse of health)	0.41	0.82	0.35	0.42	0.04
	GPS 6 (Self-abasement)	0.40	0.85	0.40	0.37	0.02
	GPS 7 (Lonely)	0.40	0.81	0.41	0.33	0.04
	GPS 8 (Dread of disease)	0.32	0.87	0.38	0.40	0.06
	GPS 9 (Attention)	−0.17	0.28	−0.01	0.17	0.10
	GPS 10 (Memory)	0.24	0.62	0.30	0.34	− 0.11
	GPS 11 (Worry of becoming a family burden)	0.30	0.65	0.50	0.27	0.03
	GPS 12 (Belief overcome disease)	0.27	0.50	0.26	0.19	−0.10
Social domain (SOD)	GSO l (Family support)	0.08	0.25	0.57	−0.07	− 0.08
	GSO 2 (Friends support)	0.19	0.20	0.63	−0.01	0.01
	GSO 3 (Family role)	0.27	0.26	0.59	0.01	−0.05
	GSO 4 (Medical assurance)	0.22	0.08	0.61	0.06	−0.08
	GSO 5 (Effects to family economy)	0.14	0.40	0.55	0.16	0.03
	GSO 6 (Effects to work/social status)	0.19	0.41	0.61	0.37	−0.08
Common symptoms and side-effects domain (SSD)	GSS l (Nausea and vomiting)	0.23	0.33	0.13	0.63	0.14
	GSS 2 (Hair loss)	0.12	0.34	0.22	0.60	0.07
	GSS 3 (Mouth ulcer)	0.07	0.13	−0.02	0.44	0.16
	GSS 4 (Pain)	0.23	0.23	0.05	0.63	0.21
	GSS 5 (Weight change)	0.28	0.31	0.23	0.57	−0.05
	GSS 6 (Diarrhea)	0.17	0.20	0.01	0.59	0.46
	GSS 7 (Fatigue)	0.24	0.34	0.13	0.74	0.24
Specific domain (SPD)	SCR 1 (Difficulty in moving bowels)	0.09	0.09	0.06	0.46	0.50
	SCR 2 (Frequent bowel movements)	0.15	0.12	0.12	0.38	0.39
	SCR 3 (Watery stools frequently)	0.25	0.15	0.07	0.35	0.37
	SCR 4 (Had blood with stools)	−0.06	0.12	0.12	0.10	0.19
	SCR 5 (Feeling the urge to move bowels without actually producing any stools)	0.14	−0.01	−0.09	0.39	0.59
	SCR 6 (Diarrhea and constipation alternately)	0.29	0.18	0.06	0.45	0.47
	SCR 7 (Pain in abdomen)	0.21	0.12	0.02	0.49	0.50
	SCR 8 (Abdominal bloat)	0.24	0.13	0.01	0.47	0.47
	SCR 9 (Pain in buttocks or waist)	0.21	0.12	−0.02	0.38	0.38
	SCR 10 (Difficulty in caring for the stoma)	0.11	0.26	−0.17	0.28	0.54
	SCR 11 (Self-abasement to because of the stoma)	−0.11	0.42	−0.09	0.21	0.43
	SCR 12 (Worry of smelling stools)	−0.04	0.39	0.02	0.24	0.43
	SCR 13 (Effects to social activity by the stoma)	0.09	0.34	0.02	0.19	0.37
	SCR 14 (Inflammation around the stoma)	−0.02	0.23	0.04	0.33	0.45

Correlation coefficients for domain scores between FACT-C and QLICP-CR are shown in Table 3. FACT-C was used to evaluate the criterion-related validity. The Pearson correlation coefficients between the specific domain of QLICP-CR and the colorectal cancer subscale of FACT-C, QLICP-GM and FACT-G, and the two overall scores were 0.37, 0.77 and 0.70. Generally, the correlations between similar domains were higher than between different domains. For example, the highest correlation coefficient for the psychological domain of QLICP-CR was with the emotional well-being domain of FACT-C, at 0.75.

**Table 3:** Correlation coefficients of domain scores between FACT-C and QLICP-CR (*n* = 152)

FACT-C	QLICP-CR						
	PHD	PSD	SOD	SSD	SPD	QLICP-GM	TOT
Physical well-being (PWB)	0.33*	0.53*	0.39*	0.45*	0.01	0.59*	0.49*
Social/family well-being (SWB)	0.21*	0.35*	0.43*	− 0.11	−0.09	0.33*	0.24*
Emotional well-being (EWB)	0.42*	0.75*	0.41*	0.35*	0.10	0.73*	0.66*
Functional well-being (FWB)	0.61*	0.50*	0.43*	0.17*	− 0.07	0.60*	0.47*
Colorectal cancer subscale (CCS)	0.47*	0.36*	0.22*	0.52*	0.37*	0.51*	0.61*
Functional Assessment of Cancer Therapy – Generic Scale (FACT-G)	0.54*	0.72*	0.57*	0.29*	− 0.02	0.77*	0.63*
Total scale (TOTAL)	0.59*	0.72*	0.55*	0.38*	0.07	0.80*	0.70*

*Correlations are statistically significant (*P* < 0.05)

**Fig. 1:**
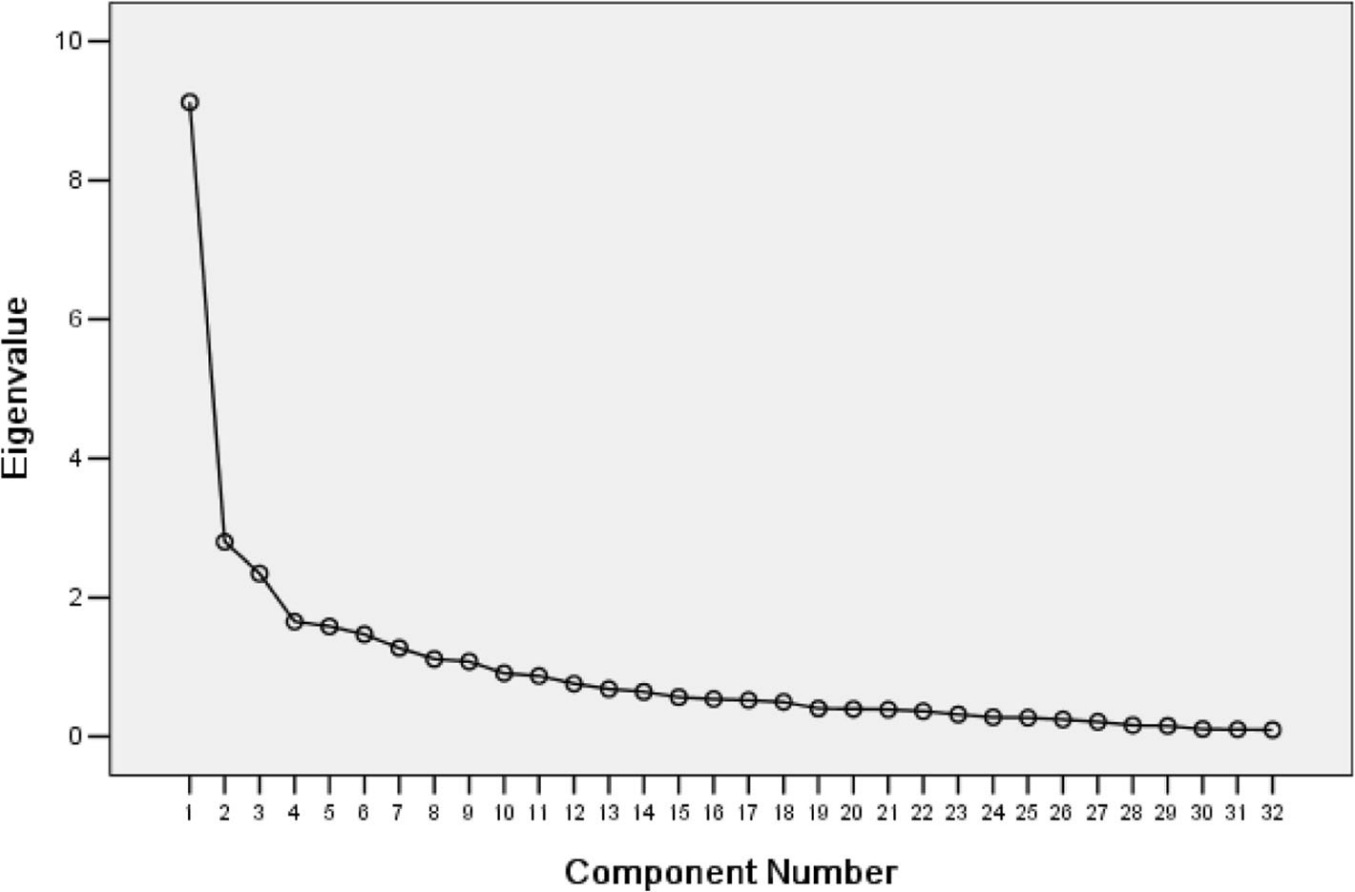
Scree plot

Principal components and the factor loadings of QLICP-GM are shown in Table 4. A scree plot was also provided, and the results are shown in Fig. 1. Based on Bartlett’s test of sphericity (χ^2^ = 2633, *P* < 0.001) and the value of the Kaiser-Meyer-Olkin test (0.84), we used EFA of QLICP-GM with varimax rotation. Using the initial eigenvalues > 1, we extracted nine principal components. Using factor loadings above 0.50, the first principal component corresponded to most items of the psychological domain (nine items, 0.62–0.89), the second to most items of the physical domain (four items, 0.53–0.86), the third with most items of the social domain (four items, 0.55–0.81), and fourth with two items from the common symptoms and side-effects domain (0.68–0.79). The fifth principal component had only one item (0.84), from the physical domain, the sixth had three (0.56–0.82) from the common symptoms and side-effects domain, the seventh had two (0.63–0.80) from the social domain, the eighth had two (0.53–0.66) from the psychological domain, and the ninth had one item (0.75) from the common symptoms and side-effects domain. Overall, these principal components accounted for 70.21% of the cumulative variance and reflected four domains of QLICP-GM. A four-factor solution (breaking at the fourth factor) could be seen on the scree-plot diagram. The variance contribution rate of each factor gradually became smaller after the fourth factor. Three principal components were extracted from the 14 items in the specific domain of QLICP-CR, accounting for 72.26% of the cumulative variance.

**Table 4:** Principal components and the factor loadings of QLICP-GM (*n* = 152)^a^

Domain	Item(brief description)	Principal components								
		1	2	3	4	5	6	7	8	9
Physical domain (PHD)	GPH l (Appetite)		0.53							
	GPH 4 (Effects to entertainment)					0.84				
	GPH 5 (Housework)		0.72							
	GPH 6 (Ability of daily living)		0.86							
	GPH 7 (Mobility)		0.74							
Psychological domain (PSD)	GPS l (Anxiety/depression)	0.84								
	GPS 2 (Dysthymia)	0.89								
	GPS 3 (Dysphoria)	0.85								
	GPS 4 (Disturbed by bad mood)	0.86								
	GPS 5 (Worry of becoming worse of health)	0.75								
	GPS 6 (Self-abasement)	0.82								
	GPS 7 (Lonely)	0.79								
	GPS 8 (Dread of disease)	0.83								
	GPS 9 (Attention)								0.66	
	GPS 10 (Memory)								0.53	
	GPS 11 (Worry of becoming a family burden)	0.62								
Social domain (SOD)	GSO l (Family support)			0.74						
	GSO 2 (Friends support)			0.81						
	GSO 3 (Family role)			0.64						
	GSO 4 (Medical assurance)			0.55						
	GSO 5 (Effects to family economy)							0.63		
	GSO 6 (Effects to work/social status)							0.80		
Common symptoms and side-effects domain (SSD)	GSS l (Nausea and vomiting)						0.56			
	GSS 2 (Hair loss)						0.73			
	GSS 3 (Mouth ulcer)						0.82			
	GSS 4 (Pain)									0.75
	GSS 6 (Diarrhea)				0.79					
	GSS 7 (Fatigue)				0.68					

^a^Factor loadings bigger than 0.50 were displayed

### Responsiveness

Responsiveness of QLICP-CR was assessed by calculating the difference between the first and third measurements, shown in Table 5. In total, 145 patients completed the third survey, and the data were used to evaluate responsiveness. There were significant differences in all domains, QLICP-GM and the total scale (*P* < 0.05) between the first and third measurements. SRM values ranged from 0.60 to 0.81 for the common symptoms and side-effects domain, specific domain, QLICP-GM and the total scale, and from 0.30 to 0.48 for the physical, psychological and social domains.

**Table 5:** Responsiveness of QLICP-CR assessed by calculating the difference between the first and third measurements (*n* = 145)

Domain	Differences (Mean ± SD)	t	*P*	SRM
Physical domain (PHD)	−3.79 ± 12.48	−3.66	< 0.001	0.30
Psychological domain (PSD)	− 3.38 ± 9.00	−4.52	< 0.001	0.38
Social domain (SOD)	− 2.93 ± 6.10	− 5.79	< 0.001	0.48
Common symptoms and side-effects domain (SSD)	−5.49 ± 8.47	−7.81	< 0.001	0.65
Specific domain (SPD)	−4.30 ± 5.33	−9.70	< 0.001	0.81
Quality of Life Instruments for Cancer Patients – General Module (QLICP-GM)	−3.85 ± 6.38	−7.26	< 0.001	0.60
Total scale (TOT)	−3.98 ± 5.19	−9.25	< 0.001	0.77

Abbreviations: *SD* standard deviation, *SRM* standardized response mean

## Discussion

QLICP-CR is a specific QOL instrument developed for Chinese patients with CRC, and takes into account the Chinese cultural background. This study examined the reliability, validity and responsiveness of QLICP-CR in a sample of 152 CRC patients being treated in a key hospital for CRC, where nearly 99% of inpatients were from Northeast China. There was very little variability in the study population, especially compared with a previous study [11] in which 110 patients provided data on admission, only 54 of whom were available at the third measurement. The representativeness of this study population suggests that the results can be generalized to the population of Northeast China.

A Cronbach’s α coefficient exceeding the 0.70 criterion was achieved in the physical, psychological, common symptoms and side-effects and specific domains, and in QLICP-GM. The value for the social domain in this study (0.62) did not meet the criterion, which is consistent with studies on patients with breast cancer and nasopharyngeal cancer [41, 42]. An α value of between 0.60 and 0.69 is, however, generally considered acceptable [43]. Research in the south of China [11] found an α value for the common symptoms and side-effects domain of 0.63, and this was considered acceptable. Although the α value for the social domain in this study did not reach 0.7, we therefore still have reason to consider that the internal consistency of QLICP-CR is acceptable. Test–retest reliability coefficients in this study were mostly above 0.80, with the exception of the common symptoms and side-effects domain, and generally better than the findings from the south of China [11]. All our findings suggest that QLICP-CR had good reliability in measuring the QOL of CRC patients in Northeast China.

The correlations between items and their own domains were strong, and the criterion of 0.40 was fulfilled for most items, except for the ninth item of the psychological domain and several items of the specific domain of QLICP-CR, which was similar to the study in the south of China [11]. This may be because each item of the specific domain evaluated CRC-specific symptoms from a different perspective, which is expected to weaken the item–domain correlation. As a result, the item–domain correlation for this domain was not as good as the others. The fourth item of the specific domain (“Have you had blood in your stools?”) had smaller coefficients with all domains. This may be because very few people paid attention to whether there was blood in their stool. Another possible reason was that the symptom can be difficult to distinguish from symptoms of other conditions [44]. QLICP-CR was therefore considered to have good convergent validity. The correlations of all items with their own domains were greater than with other domains, which was similar to the results from south China [11]. The items were therefore considered to reflect the content of their domain, showing that QLICP-CR has good discriminant validity [21]. Correlation analysis of domain scores between FACT-C and QLICP-CR also showed good criterion-related validity. Drawing on the results of the convergent validity, discriminant validity and criterion-related validity, we conclude that QLICP-CR has acceptable validity to measure the QOL of CRC patients in Northeast China.

We also used EFA to further evaluate the construct validity of QLICP-CR. Our results showed that nine and three principal components were extracted from the 32 items of QLICP-GM and 14 items of the specific domain of QLICP-CR, which was consistent with the results of the previous study [11]. Of the nine principal components, the first four corresponded to the four domains of the QLICP-GM. The item loading of the fifth to ninth principal components were also indicative (*r* > 0.5). Using the principle that an item should have a higher correlation with its own domain than with others, these items were attributed to the corresponding domains. It can be seen that nine principal components reflected nine facets under four domains of QLICP-GM. The scree-plot also indicated a four-factor solution. The number of factors was determined using the Kaiser criterion and scree-plot methods. Similarly, three principal components reflected three facets of the specific domain of QLICP-CR [11]. The EFA results therefore demonstrated that QLICP-CR has a reasonable structure. This finding further confirmed the validity of QLICP-CR in CRC patients in Northeast China.

Responsiveness is the most essential property of a measuring instrument [45], and is defined as the ability to detect a clinically-meaningful change [40]. In this study, QOL of CRC patients changed significantly after treatment. There were no significant differences in the physical and social domains in the study in the south of China [11]. This suggests that QLICP-CR is more responsive in CRC patients in northern China than in the south. The values of SRM for the physical, social, and common symptoms and side-effects domains and QLICP-GM in our study were higher than in the southern research [11], but smaller for the psychological domain (0.38), the total scale (0.77) and the specific domain (0.81). The lowest value in our study, however, still reached normal levels (SRM > 0.2) and was considered acceptable. The SRM value for the specific domain was a particularly good level (0.8). All these findings indicate that QLICP-CR had good responsiveness in CRC patients in Northeast China.

The results of this study indicated that QLICP-CR was a valid QOL scale for assessing CRC patients in Northeast China. Taken together with the results of a study in the south of China, they suggest that QLICP-CR is suitable for assessing QOL of patients with CRC in both northeast and south China. There are a number of cultural differences between northeast and south China, but this scale did not seem to be greatly affected by the differences. QLICP-CR therefore had strong stability and high credibility. In the future, it is recommended that this scale should be used when evaluating QOL in Chinese patients with CRC.

This study had some limitations. Patients were only sampled from the hospital with the highest level of diagnosis and treatment of CRC in Northeast China. We wanted to keep sampling relatively feasible and accessible in the first study in the region, so we did not focus on the lower-level hospitals. For more rigorous results, a further study covering more hospitals would be necessary.

## Conclusions

This study was the first to our knowledge to evaluate the reliability, validity and responsiveness of QLICP-CR in CRC patients in northeast China. Our results showed that the QLICP-CR was a reliable, valid and sensitive instrument to use to assess the QOL of these patients.

## Abbreviations

CCS: Colorectal cancer subscale
CI: Confidence interval
CORE: The Center on Outcomes, Research, and Education
CRC: Colorectal cancer
EWB: Emotional well-being
FACT-C: Functional Assessment of Cancer Therapy-Colorectal
FACT-G: Functional Assessment of Cancer Therapy-Generic Scale
FWB: Functional well-being
ICC: Intra-class correlation
PHD: Physical domain
PSD: Psychological domain
PWB: Physical well-being
QLICP-CR: Quality of Life Instruments for Cancer Patients-Colorectal Cancer
QLICP-GM: Quality of Life Instruments for Cancer Patients-General Module
QOL: Quality of life
SD: Standard deviation
SOD: Social domain
SPD: Specific domain
SRM: Standardized Response Mean
SSD: Common symptoms/side-effects domain
SWB: Social/family well-being
TOI: Trial Outcome Index
TOT: QLICP-CR total scale
TOTAL: FACT-C total scale
